# Supplementary material for: Mediation Effect of Musculoskeletal Pain on Burnout: Sex-Related Differences
Source: Int J Environ Res Public Health. 2022 Oct 8;19(19):12872. doi: 10.3390/ijerph191912872 (PMC9566025; doi:10.3390/ijerph191912872)
Supplement: Supplementary file 1 [file ijerph-19-12872-s001.zip › ijerph-1886601-supplementary.pdf]

# Supplementary Information

Table S1 the professional field and sex of participants

| Professional field | individuals | | | |
|---|---|---|---|---|
| | Total | % | Women | Men |
| Attending physician[1] | 71 | 4.40 | 21 | 50 |
| Resident physician[1] | 67 | 4.15 | 25 | 42 |
| Nurses | 613 | 37.96 | 587 | 26 |
| Respiratory therapist[2] | 15 | 0.93 | 14 | 1 |
| Physical therapist[2] | 35 | 2.17 | 23 | 12 |
| Social worker[2] | 11 | 0.68 | 9 | 2 |
| Nurse Practitioner[2] | 53 | 3.28 | 47 | 6 |
| Nutritionist[2] | 15 | 0.93 | 14 | 1 |
| Occupational Therapist[2] | 31 | 1.92 | 18 | 13 |
| Medical Radiation Technologist[2] | 45 | 2.79 | 25 | 20 |
| Medical technologist[2] | 75 | 4.64 | 60 | 15 |
| Psychologist[2] | 3 | 0.19 | 3 | 0 |
| Administration Staffs | 581 | 35.98 | 468 | 113 |

[1], individuals were reclassified as Physicians; [2], individuals were reclassified as professional and technical personnel
